# Calcium Hydroxylapatite‐Based Fillers in Dorsal Hand Rejuvenation: A Practical Checklist for Formulation and Protocol Specificity

**DOI:** 10.1111/jocd.71024

**Published:** 2026-07-01

**Authors:** Chuan‐Yuan Lin

**Affiliations:** ^1^ Nanzi Annyeong Clinic Kaohsiung City Taiwan; ^2^ Chengdu Royalty Aesthetic Clinic Chengdu City Sichuan Province China; ^3^ Hangzhou Royalty Aesthetic Clinic Hangzhou City Zhejiang Province China

**Keywords:** CaHA, calcium hydroxylapatite, dorsal hand rejuvenation, generic name

To the Editor,

Calcium hydroxylapatite (CaHA)‐based fillers are widely used for dorsal hand rejuvenation because they can provide immediate camouflage of tendons and veins [1, 2]. Depending on protocol, they may also improve skin quality over time [3]. However, CaHA‐based fillers are not necessarily formulation‐equivalent, with measurable differences across products and variants that can alter handling behavior [4, 5, 6]. In the thin dorsal hand envelope, such differences may translate into clinically relevant variation in spread, cohesivity, palpability, contour regularity, and tolerability [3, 4]. Accordingly, we offer a brief, practical checklist to help readers verify key formulation and protocol details when interpreting dorsal hand CaHA studies and to assist practitioners in contextualizing formulation and protocol selection for individual treatment goals.

In the dorsal hand literature, authors may describe the injected material simply as “CaHA,” even though formulation context and postmixing state may differ meaningfully across products and protocols [4, 5, 6]. In hand‐specific studies, outcomes depend on the exact injected state and technique [1, 2, 3]. For example, a randomized, blinded trial demonstrated clinically meaningful improvement in dorsal hand volume loss through 12 months using CaHA‐based fillers and standardized outcome assessment [1]. Separately, a prospective evaluator‐blinded, side‐to‐side study of diluted CaHA‐based fillers reported improvements in the Merz Hand Grading Scale and the Global Aesthetic Improvement Scale. It also showed improvements in objective parameters such as ultrasound‐based dermal measures, viscoelasticity, and collagen density [3]. These findings suggest that CaHA‐based fillers for the hands can reflect different therapeutic intents, including volumization and biostimulation, and different handling behavior [1, 3].

In parallel, contemporary CaHA‐based products and variants may not be formulation‐equivalent because they can differ in carrier matrix, CaHA loading, and postmixing handling characteristics, and CaHA–carrier systems show dilution‐ and mixing‐dependent rheologic shifts [4, 5, 6]. A previous study using carboxymethylcellulose as the carrier matrix demonstrated systematic changes in viscoelastic properties, cohesivity, and extrusion force with aqueous dilution, supporting the idea that dilution moves the material along a continuum from “filling” toward “regenerative” handling characteristics [4]. A subsequent report further differentiated dilutional rheology among Radiesse, Radiesse (+), and a lidocaine‐mixed preparation—illustrating that even within a familiar brand family, small formulation or mixing differences can produce distinct material profiles [5].

Accordingly, when interpreting dorsal hand CaHA‐based filler studies, readers and practitioners may benefit from a brief, nonjudgmental “checklist” that clarifies what was actually injected and how it was delivered. In several reports, the commercial CaHA‐based product is identified only once, often in the Methods section or Disclosures, and subsequent text defaults to the shorthand “CaHA,” [1, 2, 3] so clinicians should confirm formulation and mixing details that may influence handling, contour regularity, and tolerability [3, 4, 5, 6]. Patient variables, including baseline hand‐aging severity, tendon and vein prominence, dermal thinning, photodamage, smoking status, and prior rejuvenation procedures, should also be considered when interpreting efficacy and tolerability. First, identify the exact formulation context, including the carrier matrix, and whether a variant or lidocaine‐containing preparation was used [2, 5, 6]. Next, clarify the postmixing state: the final dilution ratio, the type of diluent, the mixing method, any resting period, and the time from mixing to injection, because these factors can shift handling characteristics [3, 4, 5]. Equally important are the delivery variables that determine product distribution in the thin dorsal hand envelope—needle versus cannula, including gage and length; entry points; injection plane; delivery technique; volume deposited per pass and per hand; distribution pattern; and whether molding, massage, or specific aftercare was performed [3]. Readers and practitioners should also note the intended treatment goal, as this often tracks with dilution, plane selection, delivery device, and endpoint timing [3, 4]. Finally, review outcome assessment methodology, including standardized photography, blinded evaluation, validated hand‐aging scales, patient‐reported outcomes, and objective measures when available, and interpret efficacy and safety by endpoint timing and adverse‐event definitions [1, 2, 3]. The checklist is summarized in Table 1.

**TABLE 1:** Practical checklist of dorsal hand “CaHA” studies and clinical applications.

Item to verify	What to check
Formulation context	Exact commercial product and formulation Carrier matrix (CMC, HA, or other carrier systems) Product variant name CaHA concentration or loading, if reported Lidocaine presence and amount
Postmixing state	Final dilution ratio or added volume Diluent type (saline, lidocaine, or other diluent) Mixing method (number of passes, mixing time, or device used) Resting period or time from mixing to injection Whether the injected product was undiluted, lidocaine‐mixed, diluted, or hyperdiluted
Patient variables	Baseline hand‐aging severity and whether a validated scale was used Degree of dorsal volume loss Tendon, vein, and bony prominence visibility Dermal thinning, skin laxity, photodamage, or actinic changes Age, smoking status, anticoagulant use, and relevant comorbidities Prior hand rejuvenation procedures Whether outcomes were stratified by baseline severity
Delivery variables	Needle versus cannula, including gage and length Entry points and number of passes Injection technique (bolus, linear threading, retrograde threading, fanning, or diffuse delivery) Injection plane (subdermal/superficial lamina, intermediate lamina, or deep lamina) Volume deposited per pass and total volume per hand Distribution pattern, molding, massage, and aftercare
Intended treatment goal	Immediate volume restoration Camouflage of dorsal veins, tendons, or bony prominences Skin‐quality improvement or dermal biostimulation Combined volumization and biostimulation Whether the stated goal explains the selected formulation, dilution, injection plane, device, and endpoint timing
Outcome assessment methodology	Standardized photography and hand positioning Blinded evaluator assessment Validated hand‐aging scales (MHGS or BHVSS)^a^ GAIS, patient satisfaction, or other patient‐reported outcome measures Objective assessments (ultrasound, viscoelasticity, profilometry, histology, or collagen analysis) Whether immediate volumization endpoints were distinguished from later remodeling or biostimulatory endpoints
Endpoint timing	Early time points that may mainly reflect carrier‐related filling or immediate camouflage Intermediate and later time points that may better reflect tissue remodeling, biostimulation, persistence, or durability Whether retreatment was allowed and how retreatment affected durability interpretation
Safety definitions and rates	How palpability, contour irregularity, edema duration, bruising, nodularity, and functional symptoms were defined Numerator and denominator for each adverse event Severity, duration, management, and resolution of adverse events Whether adverse events were reported separately for different formulations, dilutions, planes, devices, or retreatment sessions

Abbreviations: BHVSS, Busso Hand Volume Severity Scale; CaHA, calcium hydroxylapatite; CMC, carboxymethylcellulose; GAIS, Global Aesthetic Improvement Scale; HA, hyaluronic acid; MHGS, Merz Hand Grading Scale.

^a^ Examples include the MHGS, as used in prior CaHA hand studies [1, 3], and the BHVSS, as used by Busso et al. [2].

In conclusion, CaHA should be interpreted as a formulation‐plus‐protocol exposure rather than a generic category. This brief checklist may help readers translate evidence appropriately, compare outcomes fairly, and assist practitioners in contextualizing CaHA formulation and protocol selection according to patient‐specific tissue characteristics and treatment goals in dorsal hand rejuvenation.

## Conflicts of Interest

The author declares no conflicts of interest.

## Data Availability

Data sharing not applicable to this article as no datasets were generated or analyzed during the current study.
